# Understanding self-organized regularities in healthcare services based on autonomy oriented modeling

**DOI:** 10.1007/s11047-014-9472-3

**Published:** 2015-02-01

**Authors:** Li Tao, Jiming Liu

**Affiliations:** 1 Faculty of Computer and Information Science, Southwest University, Chongqing, China; 2 Department of Computer Science, Hong Kong Baptist University, Kowloon, Hong Kong

**Keywords:** Autonomy-Oriented Computing (AOC), Cardiac surgery services, Complex systems, Self-organized regularities, Patient arrivals, Wait times

## Abstract

Self-organized regularities in terms of patient arrivals and wait times have been discovered in real-world healthcare services. What remains to be a challenge is how to characterize those regularities by taking into account the underlying patients’ or hospitals’ behaviors with respect to various impact factors. This paper presents a case study to address such a challenge. Specifically, it models and simulates the cardiac surgery services in Ontario, Canada, based on the methodology of Autonomy-Oriented Computing (AOC). The developed AOC-based cardiac surgery service model (AOC-CSS model) pays a special attention to how individuals’ (e.g., patients and hospitals) behaviors and interactions with respect to some key factors (i.e., geographic accessibility to services, hospital resourcefulness, and wait times) affect the dynamics and relevant patterns of patient arrivals and wait times. By experimenting with the AOC-CSS model, we observe that certain regularities in patient arrivals and wait times emerge from the simulation, which are similar to those discovered from the real world. It reveals that patients’ hospital-selection behaviors, hospitals’ service-adjustment behaviors, and their interactions via wait times may potentially account for the self-organized regularities of wait times in cardiac surgery services.

## Introduction

A healthcare service system has been well recognized as a self-organizing system (Rouse 2008; Lipsitz 2012). Here, by the notion of self-organizing it is meant that certain forms of global order emerge without any direct control imposed from outside the healthcare service system but arise out of the local interactions between autonomous entities within the system. In the previous work, some self-organized regularities in wait times, such as the power-law distribution of variations in specialists’ waiting lists (i.e., the variations in the mean time that patients spend on specialists’ waiting lists) (Smethurst and Williams 2002), have been reported. However, it is still unclear what and how patients’ and hospitals’ behaviors with respect to underlying factors, such as distance from homes to services, hospital resourcefulness in terms of physician supply, and service performance as measured in wait times, account for such emergent regularities.

**Fig. 1:**
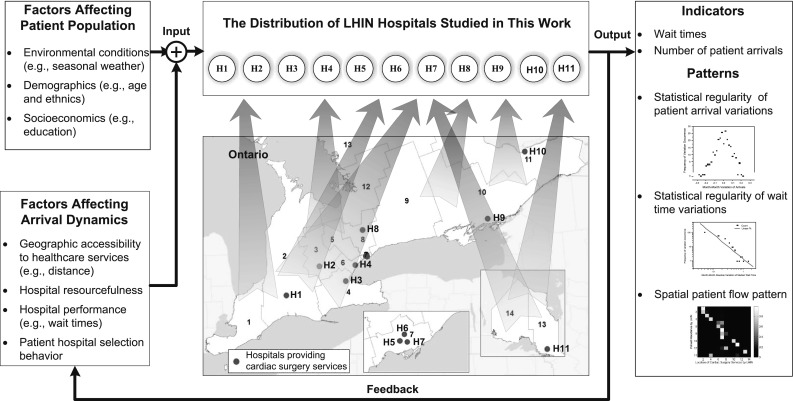
A schematic diagram of the cardiac surgery services in Ontario, Canada. Numbers in the map denote 14 Local Health Integration Networks (LHINs), which are geographic-location-based health authorities responsible for planning and determining healthcare service needs and priorities in certain areas of Ontario, Canada. H1–H11 denote the LHIN hospitals studied in this work. The illustrated tempo-spatial patterns on the right-hand side are observed from secondary data about cardiac surgery service utilization between January 2005 and December 2006. The map of Ontario was adapted from http://www.csqi.on.ca/cms/one.aspx?portalId=258922&pageId=273312

Dynamically-changing patient arrivals and wait times may be directly or indirectly affected by various factors, as schematically illustrated in Fig. 1. They include, but are not limited to, the factors of demographics, socioeconomic backgrounds, environmental conditions, as well as the healthcare related behaviors of patients (Cardiac Care Network of Ontario 2005) and hospitals (Wijeysundera et al. 2010). For instance, old age is an important risk factor for cardiovascular diseases, while patients’ hospital-selection behaviors may heavily influence the distribution of actual patient flows to various hospitals. Furthermore, these factors may have complex interrelationships and coupling interactions (Plsek and Greenhalgh 2001). For instance, as shown in Fig. 1, wait times, as one of the indicators for measuring the performance of healthcare services, may affect patients’ hospital-selection behaviors, which will in turn influence the distribution of patient flows and further exert effects on the performance of hospitals.

In view of this, to understand the self-organized regularities of patient arrivals and wait times in healthcare services from a complex systems perspective, it would be essential to address the following issues:

- *Scope* What factors, variables, processes, and hierarchical levels (e.g., services at a hospital level or at a regional level) are relevant to the self-organized regularities, and hence should be investigated and modeled?
- *Coupling relationships and/or interactions* What are the interrelationships among the impact factors and variables? Identifying their local feedback loop(s) would be crucial for understanding the self-organized regularities.
- *Heterogeneity* The behavior of patients in choosing hospitals may be heterogeneous due to the differences of personal profiles, socioeconomic backgrounds, and service availability in and around their residence areas. Hospitals may also be heterogeneous in delivering healthcare services because of variations in their equipped resources, management strategies, and dynamically-changing patient arrivals. Thus, capturing the heterogeneity of patients and hospitals will be central to the modeling and simulation of a real-world complex healthcare system.

In this paper, we present a study on applying Autonomy-Oriented Computing (AOC), an approach effective in modeling systems from a self-organizing systems perspective (Liu et al. 2004), to understand the self-organized regularities relating to patient arrivals and wait times in cardiac surgery services in Ontario. Specifically, we construct an AOC-based cardiac surgery service model (AOC-CSS model) which takes into consideration some of the key factors impacting patient arrivals (as shown in Fig. 1), i.e., weather, demographics of cities/towns in Ontario, geographic accessibility to cardiac surgery services, resourcefulness of physicians in a hospital, hospital performance in terms of wait times, patients’ hospital-selection behaviors, and hospitals’ service-adjustment behaviors. By experimenting with the AOC-CSS model, we aim to discover the underlying factors and the interactions that account for the self-organized regularities of patient arrivals and wait times.

The remainder of this paper is organized as follows. Section 2 surveys related work on modeling and simulation of wait times in a healthcare service system and briefly introduces AOC. Section 3 states the research problem and related issues. Section 4 shows the formulation of our AOC-CSS model. Section 5 presents the simulation-based studies and results on characterizing the regularities of patient arrivals and wait times. Section 6 discusses the underlying mechanism that potentially accounts for the self-organized regularities of wait times and presents a sensitivity analysis on the key parameters that influence the emergence of self-organized patterns. We finally summarize our findings and consider future work in Sect. 7.

## Related work

In general, existing studies related to uncovering the causes of the dynamics and self-organized regularities in patient arrivals and wait times can be classified into two categories: (1) those to empirically identify the effects of multiple factors based on multivariate analysis, and (2) those to characterize the behaviors of healthcare service systems. In this section, we will first review the existing studies. Then, we will briefly introduce AOC, the method that we utilize in this work to model a healthcare service system.

### Empirical identification of impact factors

Dynamically-changing patient arrivals and wait times may be affected by various factors, as schematically illustrated in Fig. 1. These factors include but are not limited to demographics, socioeconomics, environment (e.g., weather), human behaviors (Cardiac Care Network of Ontario 2005), as well as services’ physical and human resources (Wijeysundera et al. 2010). A number of previous studies have aimed to discover the underlying factors and estimate the corresponding impacts on patient arrivals and wait times from empirical data.

To achieve this end, most of the existing studies rely on multivariate analysis methods to unveil the potential impact factors and the corresponding effects. Among those methods, factor analysis has been commonly utilized to extract various underlying factors from a set of observed variables, such as those contributing to long wait times (Pillay et al. 2011). Various multiple regression methods (Knapman and Bonner 2010), especially multiple linear regression (Hair et al. 1998) and logistic regression (Menard 2009), has been extensively employed to analyze pairwise relationships between observed factors and patient arrivals or wait times (Sanmartin et al. 2007). Recently, structural equation modeling has drawn increasing attentions in healthcare service research for it enables us to investigate a series of complex (direct and indirect, pairwise and hierarchical) relationships among observed and latent (i.e., not directly measured) variables simultaneously (Hair et al. 1998).

Aided by these methods, existing studies have identified several factors and the corresponding effects on patient arrivals and wait times. For instance, some studies have found that geodemographic profiles, including population size (Buerhaus et al. 2009), age profile (Grover et al. 2009), and socioeconomic characteristics (Smith et al. 2009), have significant effects on patient arrivals to different hospitals. Tao et al. (2013) have further found that certain geodemographic profiles, such as geographic accessibility as measured by the distances from homes to services, may moderate the relationship between population size and patient arrivals, as well as the relationship between age profile and patient arrivals. As another example, researchers have found that the available physical (e.g., operating rooms and beds) (Cardiac Care Network of Ontario 2006) and human resources (e.g., skilled doctors, nurses, and anesthetists) (Wijeysundera et al. 2010) of healthcare services, as well as management policies (Jun et al. 1999) are significantly related to wait times.

Although the studies of empirically analyzing impact factors can reveal the reason why patient arrivals and wait times change to some extent, such studies cannot explain how healthcare services self-regulate in terms of patient arrivals and wait times because (1) these studies assume that the relationships between factors and patient arrivals or wait times do not change; (2) the employed multivariate analysis methods do not intend to characterize the behaviors of a healthcare service system, and thus they cannot represent the dynamics of patient arrivals and wait times.

### Characterization of systems behaviors

In healthcare, patient arrivals and wait times dynamically change over time and differ from one hospital to another. To characterize the dynamics of patient arrivals and wait times, studies utilize various modeling and simulation methods including stochastic modeling and simulation, system dynamics, agent-based modeling, and AOC, to model the behaviors of a healthcare service system from different aspects.

Stochastic modeling and simulation methods, such as queueing theory (Kleinrock 1975) and discrete event simulation (England and Roberts 1978), are commonly used methods to model and simulate a healthcare service system by describing its stochastic properties. Models based on these methods aim to estimate probability distributions of potential states (e.g., as represented by the queue length) of a system, as referred to as regularities in this paper, by taking into account the variations in one or more variables. In healthcare services research, previous studies have utilized queueing models and discrete event simulations to analyze the change of waiting lists for designing a specific healthcare service system (Fomundam and Herrmann 2007); to present the dynamics of operating rooms and recovery rooms under the constraints of capacity (e.g., beds and recovery time) (Schoenmeyr et al. 2009; Creemers and Lambrecht 2008); and to predict the performance of a healthcare service system in different scenarios (Fomundam and Herrmann 2007). To summarize the applications of queueing models and discrete-event simulations in healthcare services research, Jun et al. (1999), Fone et al. (2003), and Jacobson et al. (2006) have presented comprehensive surveys on the use of the two methods to address various problems such as forecasting the dynamics of patient flows with different resource allocation strategies. Based on these reviews, in 2010, Cardoen et al. (2010) have incrementally reviewed the up-to-date studies that employ the two methods for operating room planning, scheduling, and performance modeling. Despite the wide applications in healthcare, these methods assume the existence of passive entities in the system, which makes it difficult to model entities’ autonomous behavior with respect to certain impact factors. Therefore, these methods cannot explain how self-organized regularities in wait times emerge from individuals’ behaviors and interactions.

In addition, researchers have developed various models based on system dynamics to understand the dynamically-changing behaviors of a healthcare service system with a focus on the internal feedback loops. System dynamics is distinct from other methods in that it utilizes variables (as referred to as stocks) and the corresponding interactions (as referred to as flows) between each other (Maani and Cavana 2000) to model a system as a causal loop diagram. In healthcare services research, studies have employed this method to qualitatively characterize the effects of interrelated impact factors and wait times in the cardiac care system of Ontario, Canada (Cardiac Care Network of Ontario 2006); model the relationships between multiple interacting diseases, healthcare service systems for delivery corresponding services, and national and state policies (Homer and Hirsch 2006); simulate patient flows with the purpose of identifying bottle-necks in emergency healthcare (Brailsford et al. 2004); and predict the demand for ambulatory healthcare services (Diaz et al. 2012). However, system dynamics may be hard to address the problem of explaining the causes of self-organized regularities because: (1) the assumption that entities contained in a stock are homogeneous makes this method be hard to model patients’ heterogeneous behaviors in selecting hospitals; (2) the predefined and fixed interactions between stocks do not allow this method to model patients’ and hospitals’ autonomous behaviors.

Furthermore, a majority of studies have employed the method of agent-based modeling to model a healthcare service system through describing the behaviors and interactions of autonomous individuals (Grimm and Railsback 2005) (as referred to as agents, which could be either a physical element such as a patient, or an abstract concept such as a hospital). In ABM, each agent makes decisions individually according to its behavioral rules and perceived environmental information (Wooldridge 2009). Each agent may also interact with each other by means of competition, cooperation, or environmental information sharing. Because of the features of autonomy and interaction, even a simple agent-based model may emerge specific regularities or patterns at a systems level (Bonabeau 2002; Epstein 2006).

Based on ABM, researchers have built models for different research purposes, such as for examining the effects of physicians’ behaviors on patient outcome (Leykum et al. 2012); predicting the spread of infectious diseases based on social networks (Ajelli et al. 2010; Eubank et al. 2004); and evaluating patient scheduling or other operation management strategies (Barnes et al. 2013). However, traditional ABM faces two major challenges in characterizing system-level self-organized regularities: (1) it lacks general principles to explicitly indicate which fundamental behaviors of and interactions between agents play crucial roles in the emerging patterns and therefore should be modeled; (2) it does not emphasize the identification and the modeling of feedback loops in a system. Potentially due to this reason, some of the existing models based on ABM appear to be more or less ad hoc with a major focus on delicately defining agents, whereas few of them pay attention to explaining the underlying mechanisms for self-organized regularities in a healthcare service system.

### Autonomy-oriented computing

AOC (Liu 2008) is a computational modeling and problem-solving paradigm with a special focus on addressing the issues of self-organization and interactivity by modeling heterogeneous individuals (entities), autonomous behaviors, and the mutual interactions between entities and certain impact factors. Compared with ABM, AOC is more practical for discovering the underlying mechanisms for emergent patterns, as AOC provides a general principle, i.e., *AOC-by-prototyping* (Liu 2001), for explicitly stating what fundamental behaviors of and interactions between entities should be modeled. Generally speaking, AOC-by-prototyping includes the following three key steps to model a system (Liu et al. 2004):

- *Identify entities, key impact factors, and local feedback loops* In the first step, autonomous entities, the key impact factors, the mutual interactions between entities and factors, and the local feedbacks loops that may play significant roles in the self-organization of the system should be recognized based on the literature and the observations of the interested system.
- *Identify environment characteristics and define environment E* In the second step, the types of information that are collected and exchanged in the environment should be determined. Accordingly, the environment that entities reside in and interact with should be formally modeled.
- *Define entities, autonomous behaviors, and behavioral rules* This step handles the modeling and the design of local-autonomy-oriented entities, their autonomous behaviors, and behavioral rules. This step needs to clearly state how entities react with respect to different impact factors and respond to various information; and how entities directly interact with or indirectly interact via sharing information in the environment, with a special attention on how the interactions form positive or negative feedback loops.

AOC-by-prototyping should be an evolutionary and exploratory process (Liu et al. 2004) to make the synthetic system as real-world driven as possible. During this process, some parameters are initialized and configured to make the synthetic model approximate the real system more closely. The final synthetic model can be used to reveal the underlying mechanisms of positive-feedback-based aggregations or negative-feedback-based regulations, which may account for the observed self-organization and emergent behavior of the real system.

Aided by AOC, Tao and Liu (2013) have revealed that the hospital-selection behavior of patients and the interaction between this behavior and hospital wait times may account for the self-regulating service utilization in a cardiac care system. However, in the reported AOC-based model, the assumption that patients residing in a specific location are homogeneous in choosing hospitals (e.g., all the patients living in a city only consider distances from homes to hospitals when they select hospitals) seems not reasonable in the real world. Furthermore, the assumption that the average service rates of hospitals are not changed during the service processes may not be always hold in the real-world healthcare services. In this regard, we aim to develop a new AOC-based model that relaxes the two unrealistic assumptions for the purpose of characterizing the self-organized regularities of patient arrivals and wait times.

## Problem statement

In this work, as aided by AOC, we will build an AOC-CSS model to explain how certain global-level self-organized regularities emerge from the individual-level behaviors and interactions. The specific AOC-CSS model not only considers the input and the output of real-world cardiac surgery services, but also addresses the underlying interaction mechanisms among the involved heterogeneous entities, such as patients and hospitals.

**Fig. 2:**
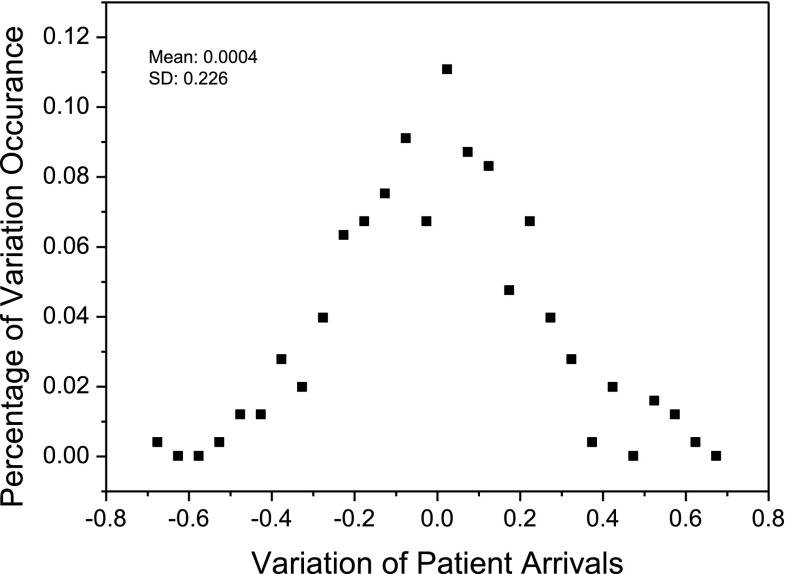
The statistical distribution of variations in patient-arrival for cardiac surgery services in Ontario, Canada, between January 2005 and December 2006. The distribution follows a normal distribution with a mean value of 0.004 and a standard deviation (SD) of 0.226. The normality of the distribution passed the Lilliefors test (*p* = 0.05)

**Fig. 3:**
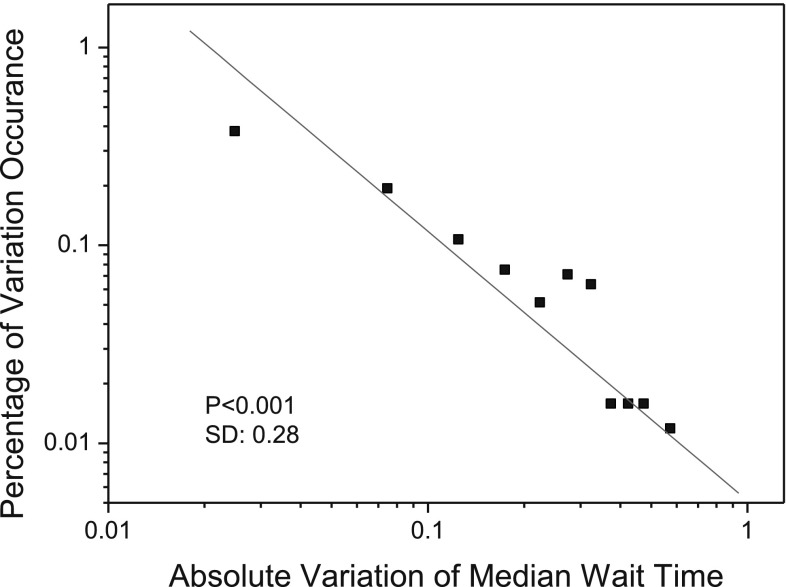
The statistical distribution of absolute variations in median wait time for cardiac surgery services in Ontario, Canada, between January 2005 and December 2006. The distribution follows a power law with a power of −1.36 (power-law test based on Clauset’s method (Clauset et al. 2009):* p* < 0.1; linear fitness (*red line*):* p* < 0.001; standard deviation SD = 0.28). (Color figure online)

Figures 2 and 3 illustrate the two self-organized regularities that are identified from the aggregated data about patient arrivals and median wait time for 11 hospitals providing cardiac surgery services in Ontario, Canada, over a 2-year period from January 2005 to December 2006 (the investigated data was provided by the organization of Cardiac Care Network of Ontario^1^; accessed in February 2011). As shown in Figs. 2 and 3, the monthly variations in patient arrivals follow a normal distribution based on the Lilliefors test (Sá 2007) (*p* = 0.05); while the monthly absolute variations of median wait time follow a power-law distribution with a power of −1.36 and a standard deviation of 0.28 (*p* < 0.001). In the two figures, the month-to-month variations in patient arrivals (or median wait time) are calculated as follows:

1 $$v_{n+1}=\frac{x_{n+1}-x_{n}}{x_{max}-x_{min}} \quad (n\ge 1)$$

where $v_{n+1}$ denotes the variation of patient arrivals (or median wait time) at time* n* + 1. In this work, each* n* corresponds to a month.* x*_*n*_ denotes the number of patient arrivals (or median wait time) at month* n*.* x*_*min*_ and * x*_*max*_ are the minimum and the maximum values of patient arrivals (or median wait time) over the two-year period, respectively.

Accordingly, the absolute month-to-month variations in patient arrivals (or median wait time), $v_{n}^{\prime }$, can be obtained as follows:

2 $$v_{n}^{\prime }=|v_n| \quad (n>1)$$

Specifically, in designing the AOC-CSS model to explain the two emergent regularities as shown in Figs. 2 and 3, we will address the following issues:

- *Major impact factors* What factors, by and large, affect the patient hospital-selection behavior?
- *Behavioral rules* How to formulate the behavioral rules that govern the hospital-selection behavior, while taking into account the identified impact factors and the heterogeneity of patients and hospitals?
- *Local interactions and feedback loop(s)* What are the local interactions and feedback loop(s) of the entities in the system?
- *Simulation-based validation* Do certain self-organized regularities emerge from the AOC-CSS model based simulations?

In what follows, we will describe in detail how we build the AOC-CSS model and address the above-mentioned issues.

## An AOC-based cardiac surgery service model (AOC-CSS)

As a case study to understand self-organized regularities by means of AOC-based modeling and simulation, we present the following three steps in constructing an AOC-based cardiac surgery service model (AOC-CSS model):

1. Identifying the participating heterogeneous entities in the system, major impact factors, and local feedback loop(s).
2. Modeling the services based on the AOC methodology where a special attention is paid to deriving entities’ behavioral rules that incorporate (1) the heterogeneity of the entities, (2) the identified impact factors, and (3) the local feedback loop(s).
3. Capturing the self-organized regularities by means of simulating the constructed AOC-CSS model.

### Identifying entities, impact factors, and local feedback loop(s)

#### Entities

In Ontario, each location (e.g., a city or a town) has a certain number of patients that require cardiac surgery services. According to the “cardiac care patient access management process”^2^, when these patients are recommended to have cardiac surgery by their GPs or specialists, they will choose a specific hospital to receive the required services. In most cases, patients make their decisions with their GPs, as 93 % of Ontario’s population are registered with a GP (Ontario Ministry of Health and Long-Term Care 2012) and most of the patients will follow a GP’s recommendations (Cardiac Care Network of Ontario 2005). Patients’ hospital-selection behaviors therefore represent the consequences of patient-GP mutual decisions. After patients make a decision on hospital selection, GPs refer patients to the selected hospitals, where patients wait to receive the treatment. Finally, patients leave the hospital after finishing the treatment.

Based on the above process, we can readily identify three types of autonomous entities in the cardiac surgery services; they are: *GP*, *patient*, and *hospital*. For each patient entity, he/she and his/her GP will make a mutual decision on hospital selection based on (1) the released information about the hospitals and (2) the applicable behavioral rules for hospital selection which take into account certain impact factors.

#### Major impact factors

According to the literature, we consider the following factors that affect the patient behaviors in selecting hospitals: (1) geographic distance (between homes and a hospital), (2) the resourcefulness of a hospital (referred to as hospital resourcefulness thereafter), and (3) hospital performance (e.g., wait times). First of all, it has been well recognized that the geographic distance is negatively associated with the probability that patients and GPs select a hospital (Seidel et al. 2006; Lakha et al. 2011). The resourcefulness of a hospital, as represented by the number of physicians (Wijeysundera et al. 2010), has been found to be positively correlated with the probability that patients and GPs select a specific hospital (Wijeysundera et al. 2010; Kinchen et al. 2004; Tao and Liu 2012) because more hospital resources may attract more patient arrivals (Smethurst and Williams 2002). In addition, waiting is also a major concern for patients (Cardiac Care Network of Ontario 2005) and GPs (Lakha et al. 2011; Wakefield et al. 2012), who are usually in favor of hospitals with short wait times (Cardiac Care Network of Ontario 2005; Lakha et al. 2011; Wakefield et al. 2012).

#### Local feedback loops

**Fig. 4:**
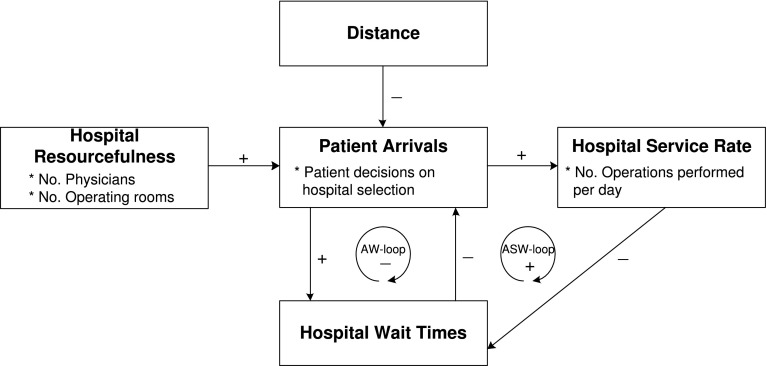
The effects of impact factors on patient-GP mutual decisions on hospital selection and the interacting feedback loop.* +* positive relationship between two factors,* –* negative relationship between two factors

The aforementioned interrelationships among these factors, patient-GP mutual decisions on hospital selection, and hospital service-adjustment behavior may form a few feedback loops. In this study, we identify two local feedback loops among patient arrivals, the service rate of a hospital, and hospital wait times (as shown in Fig. 4). The first negative feedback loop, namely as AW-loop, exists between the factors of patient arrivals and wait times due to the patient-GP mutual decisions on hospital selection. The AW-loop shows that the increase of wait times in a hospital may decrease the number of patient arrivals subsequently, as patients/GPs are less willing to select a hospital with long wait times, and thus the wait times in the hospital will be reduced soon afterwards. That means, the AW-loop will regulate the variable of patient arrivals or wait times to its original value when either of the two variables is changed. As shown in Fig. 4, the factors of patient arrivals, hospital service rate, and wait times form the second positive feedback loop, namely as ASW-loop. The ASW-loop will accelerate the changes of patient arrivals, hospital service rate, or wait times. Taking one scenario as an example, if there are more patient arrivals at a hospital, the hospital will increase its service rate to cope with the arrivals and avoid long wait times. Whereafter, the increased service rate will shorten wait times, which will in turn result in a larger number of patient arrivals afterwards.

In what follows, we will describe the detailed formulation of the AOC-CSS model, which includes the environment, the three types of entities, and their behavioral rules.

### Environment

Patients are geographically distributed in different cities and towns. The relationship between cities and hospitals can be conceptualized as a weighted bipartite network defined as follows:

#### **Definition 1**

(*City-hospital network*) A city-hospital network can be described as a bipartite network $CH=(C,H,D)$, where $C(N)=\{c_i\}\ (i\in [1,N])$ and $H(M)=\{h_j\}\ (j\in [1,M])$ are two node sets, $H\cap C=\emptyset$; $D=\{d_{ij}\}\ (i\in [1,N], j\in [1,M])$ is a set of weighted edges.

Here, each node $c_i\ (\forall c_i\in C)$ in a city-hospital network* CH* represents a sampled city/town. Each node $h_j\ (\forall h_j\in H)$ in* CH* denotes a hospital that provides cardiac surgery services in Ontario, Canada. Finally, each weighted edge $d_{ij}\ (\forall d_{ij}\in D)$ in* CH* represents the driving time from a city/town $c_i\ (\forall c_i\in C)$ to a hospital $h_j\ (\forall h_j\in H)$ which is estimated by using the “Get directions” function in Google Maps.^3^

The environment* E* in the AOC-CSS model records the released information about hospitals. We formally define environment* E* as follows:

#### **Definition 2**

(*Environment*) Environment* E* for the AOC-CSS model is represented as a city-hospital network* CH*.* E* maintains information that could be accessed by patients and GPs. We define environment* E* as a tuple: <D,R,W,POP,M>, where the elements are given as follows:

- *D* Distance information $D=\{d_{ij}\}$. Each $d_{ij}$ records the driving time between city/town $c_i$ $(\forall c_i\in C)$ and hospital $h_j$ ($\forall h_j\in H)$.
- *R* Hospital resourcefulness information $R=\{r_j\}$, where $r_j$ records the number of physicians in $h_j$ ($\forall h_j\in H)$.
- *W* Wait time information $W=\{w_j(\tau )\}$. Each $w_j(\tau )$ records the wait time information (e.g., median wait time in this paper) for hospital $h_j$ ($\forall h_j\in H)$ at time round $\tau$. Here, a unit of time round equals to $\hat{\tau }$ number of time steps, i.e., $\tau =\hat{\tau }*t$, where $\hat{\tau }$ is a positive integer, and* t* denotes a time step, e.g., one day in this paper.
- $POP$: Population information $POP=\{pop_i\}$. Each $pop_i$ records the population size for city $c_i$ $(\forall c_i\in C)$.
- *M* Patient-generation probability $M=\{m_i\}$. Each $m_i$ records the patient-generation probability for city $c_i$ $(\forall c_i\in C)$ in a time step.

### Entities

#### General physician (GP)

In the AOC-CSS model, patients come to a hospital that is selected by patient-GP mutual decisions based on the released information in the environment* E*. As most cardiac surgery patients are referred by GPs, we define entities* GP*[*N*] to record and represent patient-GP mutual decisions on hospital selection for cities/towns* C*(*N*), as given below:

##### **Definition 3**

(*General physician (GP) entity*) Each GP entity $GP[i]\ (i\in [1,N])$ records the information about the number of patients who live in city* c*_*i*_ and are referred to hospitals at time step* t*. Each entity $GP_i\ (i\in [1,N])$ maintains a record: $<cityID,\,A_{k}(t)>$, where the elements are given as follows:

- $cityID$: This represents the unique identity of a location.
- *A*_*k*_(*t*) This denotes the patient flow information for urgent type $k\ (k\in K)$ patients, $A_k(t)=\{\hat{a}_{k,j}(t)\}$. Each $\hat{a}_{k,j}(t)$ records the number of type $k\ (k\in K)$ patients to hospital $h_j$ ($h_j\in H$) at time step* t*.

#### Patient

As reported in (Cardiac Care Network of Ontario 2005), a large number of patients may not have access to wait time information and thus they may not consider wait times when they select a hospital. Patients can therefore be categorized as *wait time-sensitive* or *wait time-insensitive*, according to their decision making styles. *Wait time-sensitive* patients refer to those who consider all of the acquired information about the hospitals (i.e., distance, hospital resourcefulness, and wait times). *Wait time-insensitive* patients refer to those who do not take into account the factor of wait times when he/she selects a hospital, and those who do not know wait time information. A patient entity is defined as described below.

##### **Definition 4**

(*Patient entity*) A patient entity maintains a record: ${<}patientID, cityID, P_r, rule, hospitalID, type, joinTime, endTime, {\tilde{w}}\!{>}$, where the elements are given as follows:

- *patientID* This records the unique identity which is represented by a constant for a patient.
- *cityID* This denotes the unique identity for the city/town that a patient comes from.
- *P*_*r*_ This denotes the probability of a patient considering the factor of wait times when selecting a hospital. Accordingly, the probability of a patient who does not take into account the factor of wait times when choosing a hospital is 1 − *P*_*r*_.
- *rule* This represents how a patient chooses a hospital along with the GP.
- *hospitalID* This indicates the unique identity for the hospital that a patient arrives at.
- *type* This represents the urgent type of a patient entity to the cardiac surgery service according to the severity of illness, $\forall k\in [1,K] \ (K\ge 1)$.
- *joinTime* This denotes the time step at which a patient joins in the queue of a hospital.
- *endTime* This indicates the time step at which a patient is served in a hospital.
- $\tilde{w}$ This records how long a patient has waited in a hospital, $\tilde{w}=endTime-joinTime$.

#### Hospital

Whenever patient entities register at hospitals that are selected based on patient-GP mutual decisions, they will be arranged to different positions in the waiting queue according to their urgent types. Hospitals stochastically serve the queueing patients with different mean service rates that depend on the physical resources, human resources, and service management policies of hospitals. The mean service rate of each hospital may be regularly (e.g., once per month) adjusted in accordance with the accumulated number of patient arrivals. In other words, if there are more patients waiting in the queue, the hospital may increase the service rate, and vice versa. To model the operations of a hospital, this work employs queueing theory, which is commonly utilized by some previous studies (Schoenmeyr et al. 2009; Creemers and Lambrecht 2008). As operating rooms for cardiac surgery services in a hospital are, to a certain extent, homogeneous in terms of the service capacity, and are centrally scheduled, the operating rooms in a hospital behave like a single one. We assume that the service rate of a hospital follows an exponential distribution, which is a common assumption made by previous work (Schoenmeyr et al. 2009; Creemers and Lambrecht 2008). Thus, we can model each hospital as an M/M/1 queue (Kleinrock 1975). A hospital entity can be defined as follows:

##### **Definition 5**

(*Hospital entity*)* Hospital*[*M*] records the information of all the hospitals. Each hospital entity* h*_*j*_ ($\forall h_j\in H$) maintains a record: ${<}hospitalID, cityID, \tilde{A}_{k}(t),\mu (t), rule, w(\tau ), queue\!{>},$ where the elements are given as follows:

- *hospitalID* This represents the unique identity for a hospital.
- *cityID* This indicates the unique identity for the city/town in which a hospital is located.
- $\tilde{A}(t)_{k}$ This records the patient arrival information for type $k\ (k\in K)$ patients, $\tilde{A}(t)_k=\{\tilde{a}_{i,k}(t)\}$. Each $\tilde{a}_{i,k}(t)$ records the number of type $k\ (k\in K)$ patients coming from city/town* c*_*i*_ at each time step.
- *μ*(*t*) This denotes the hospital service rate at time step* t*.
- *rule* This represents how a hospital adjusts the service rate with respect to the accumulated patient arrivals.
- $w(\tau )$ This records the wait time information (mean median wait time in this paper) of hospital* h*_*j*_ at time round $\tau$, which will be released in environment* E*.
- *queue* This records the information about the queue, which includes all the patient entities waiting for cardiac surgery services at each time step.

### Designing behavioral rules

#### Behavioral rules for patients to select hospitals

Based on the literature review, we identify stylized facts addressing the effects of key impact factors (i.e., distance, hospital resourcefulness, and wait times) on patient-GP mutual decisions for hospital selection.

- *Stylized fact 1* The probability that patients select a hospital is exponentially and inversely related to the distance between their homes and a hospital (Seidel et al. 2006).
- *Stylized fact 2* Patients usually prefer to visit a hospital that is resourceful in terms of personnel (e.g., physicians) and facilities (e.g., ORs) (Wijeysundera et al. 2010; Kinchen et al. 2004; Tao and Liu 2012). Hospital resourcefulness and the number of patient arrivals are therefore positively correlated (Liu et al. 2011).
- *Stylized fact 3* Patients usually prefer to visit a hospital with shorter wait times (Lakha et al. 2011; Cardiac Care Network of Ontario 2005; Wakefield et al. 2012). However, a large proportion of patients, especially the elderly, may not have access to wait time information or are less likely to consider the wait times when they select hospitals (Cardiac Care Network of Ontario 2005).

Based on the stylized facts, we develop two specific behavioral rules, i.e., a DHW-rule and a DH-rule, to model how patients choose a hospital. The two behavioral rules are our assumptions in this work, which are defined below.

##### **Definition 6**

(*DHW-rule*) DHW-rule represents how a wait time-sensitive patient residing in location $c_i\ (\forall c_i\in C)$ estimates the arrival probability $a_{ij}$ for hospital $h_j\ (\forall h_j\in H)$ based on the information about distance $d_{ij}$, the hospital resourcefulness* r*_*j*_, and the released wait time information $w_j(\tau )$ at time round $\tau$. The hospital selection probability for a hospital* h*_*j*_ can be calculated as follows:

3 $$ \begin{aligned}a_{ij}&= f(d_{ij})*f(r_j)*f(w_j(\tau )) \\ f(d_{ij})&= \frac{d'_{ij}}{\sum _{h_k\in H}d'_{ik}} \\ d'_{ij}&= \frac{\sum _{h_k\in H}d_{ik}^{\alpha _d}}{d_{ij}^{\alpha _d}}\\ f(r_j)&= \frac{r_j^{\alpha _r}}{\sum _{h_k\in H}r_k^{\alpha _r}} 
\\f(w_j(\tau ))&= \frac{\sum _{h_k\in H}w_j^{\alpha _w}(\tau )}{w_{j}^{\alpha _w}(\tau )}, \end{aligned}$$

where* α*_*d*_ ($\alpha _d\in [1,5]$), * α*_*r*_ ($\alpha _r\in [1,5]$), and * α*_*w*_ ($\alpha _w\in [1,5]$) are exponents to indicate the sensitivity of patients to the factors of distance, hospital resourcefulness, and wait times, respectively.

##### **Definition 7**

(*DH-rule*) DH-rule indicates how a patient chooses a hospital* h*_*j*_ with respect to the distance $d_{ij}$ and hospital resourcefulness $r_j$. The hospital selection probability is calculated by:

4 $$\begin{aligned} a_{ij} &= f(d_{ij})*f(r_j) \\ f(d_{ij})&= \frac{d'_{ij}}{\sum _{h_k\in H}d'_{ik}} \\ d'_{ij}&= \frac{\sum _{h_k\in H}d_{ik}^{\alpha _d}}{d_{ij}^{\alpha _d}}\\ f(r_j)&= \frac{r_j^{\alpha _r}}{\sum _{h_k\in H}r_k^{\alpha _r}}\end{aligned} $$

#### A behavioral rule for hospitals to adjust service rates

**Fig. 5:**
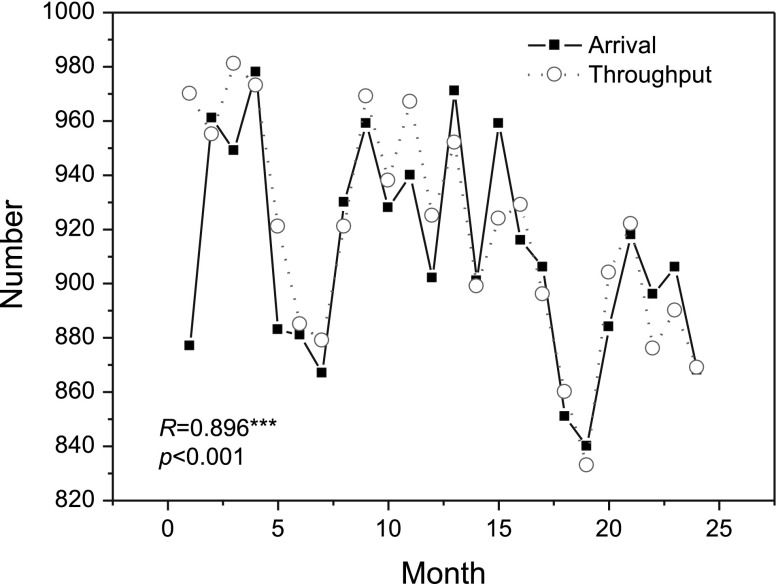
The number of patient arrivals versus the number of treated cases of cardiac surgery services in Ontario, Canada, between January 2005 and December 2006

Hospitals may periodically change their service rates to adapt to unpredictable patient arrivals. For instance, as shown in Fig. 5, changes in the throughput, which represents the actual serviced numbers of patients, follows approximately the same pattern as changes in the patient arrivals in cardiac surgery services in Ontario. The correlation coefficient between the throughput and patient arrivals is 0.896 (*p* <0.0001), implying that the service rate of a hospital may vary in accordance with the changes in patient arrivals. We therefore define an S rule for hospitals to adjust their service rates by assuming that service rate of a hospital and the queue length (representing the accumulated patient arrivals at present) is positively and linearly related. The definition of the S rule is given as below.

##### **Definition 8**

(*S-rule*) S-rule represents how a hospital $h_j\ (\forall h_j\in H)$ changes the service rate $\mu _j(t)$ in view of the aggregated patient arrivals in the past $\tilde{\tau }$ number of time steps. The service rate is updated as follows:

5 $$\mu _j(t)=\bar{\mu }_j*\left(\frac{{b_j*\sum _{t'=t-\tilde{\tau }}^{t-1} \tilde{A}_j(t')}}{\tilde{\tau }*\bar{A}_j}+g_j\right),$$

where $\tilde{\tau }$ is the number of time steps that a hospital adjusts its service rate once (usually 1 week in Ontario (Office of the Auditor General of Ontario 2009); $\mu _j(t)$ is the service rate of hospital* h*_*j*_ at time step* t*; $\bar{\mu }_j$ is the average service rate of hospital* h*_*j*_ at a time step; $A_j(t^{\prime})$ is the total number of patient arrivals at time step $t^{\prime}$; $\bar{A}_j$ is the average patient arrivals to hospital* h*_*j*_ at a time step; * b*_*j*_ ($b_j\in [0,1]$) and* g*_*j*_ ($g_j\in [0,1]$) are two parameters to represent how a hospital adjusts its service rate with respect to the variations of patient arrivals.

## AOC-CSS model based simulations

**Fig. 6:**
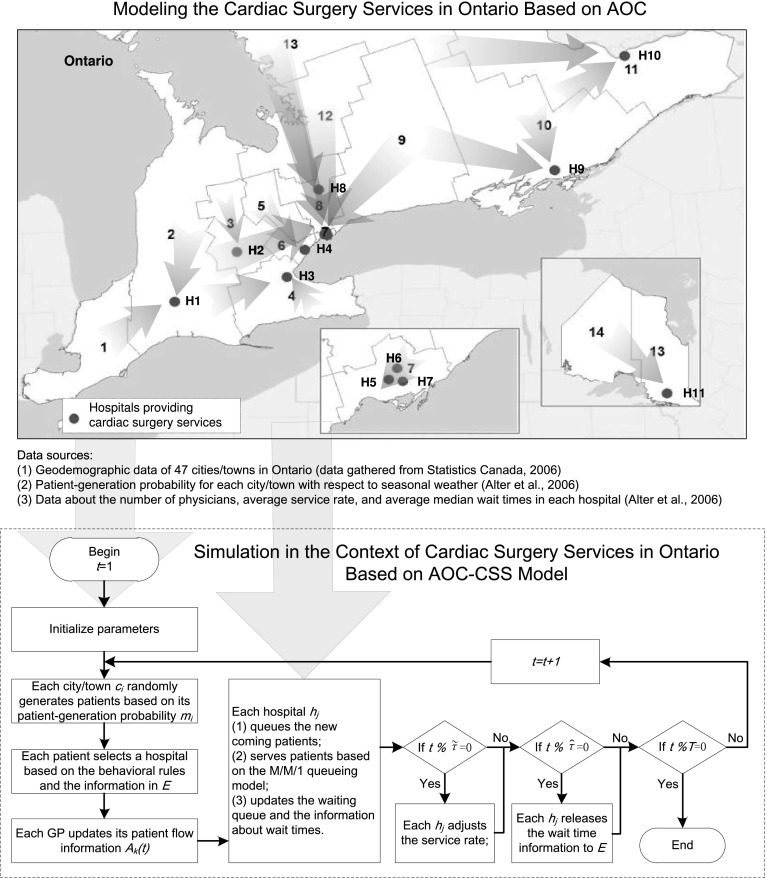
A schematic diagram to illustrate the simulation framework within the context of cardiac surgery services in Ontario, Canada. Numbers in the map denote 14 LHINs. H1 to H11 denote hospitals under LHINs. The map of Ontario was adapted from http://www.csqi.on.ca/cms/one.aspx?portalId=258922&pageId=273312

In this section, we conduct simulations based on our AOC-CSS model, aiming to understand the self-organized regularities of patient arrivals and wait times (as presented in Figs. 2 and 3) in cardiac surgery services in Ontario, Canada. The overall simulation framework is schematically illustrated in Fig. 6.

As presented in Fig. 6, at each time step, a simulated city/town* c*_*i*_ in Ontario randomly generates a certain number of patient entities based on the mean patient size $pop_i*m_i$. Each generated patient entity in * c*_*i*_ calculates the arrival probability for each hospital based on the behavioral rules, and then selects a hospital with its GP. At the same time, each* GP*_*i*_ calculates the total number of patient entities coming from city* c*_*i*_ to each hospital. Then, each hospital entity* h*_*j*_ queues the coming patient entities and services them accordingly. The service time for a specific patient entity in * h*_*j*_ is randomly generated from an exponential distribution with the mean service rate* μ*_*j*_. Furthermore, at each time round (e.g., at each month in this work), a hospital entity * h*_*j*_ should calculate its wait time information and release it to environment* E*. Specifically, within the research scenario, we simulate cardiac patients coming from 47 major cities/towns (each has a population of more than 40,000 in 2006) in Ontario, Canada, for which cover approximately 90.72 % of Ontario’s total population. We also simulate 11 hospitals that provide cardiac surgery services in Ontario.

### Simulation settings

The parameters in the AOC-CSS model are initialized using aggregated data which is published by Cardiac Care Network of Ontario (CCN) of Ontario and 2006 Canada Census (Statistics Canada 2007). CCN published monthly statistical reports on cardiac surgery service utilization in Ontario hospitals in the years between January 2005 and December 2006 (we accessed the data in February 2011). In the statistical reports, the average number of treated cases, the median wait time, and the queue length in a month for each hospital were reported. Therefore, the service rate* μ*_*j*_ can be approximated as the average number of served cases in a day. The service rate adjustment parameters* b*_*j*_ and* g*_*j*_ for hospital* h*_*j*_ can also be estimated based on the CCN data.

To estimate the arrival rate for a hospital in a day, we calculated the number of patients in each city/town by multiplying the patient-generation probability, i.e., the probability of a person in a city/town to be a patient who needs a cardiac surgery service, to the total number of people in the city/town. In this work, the patient-generation probability for each city/town could be inferred from the work of Alter et al. (2006). The total population for each city/town is gathered from the 2006 Canada Census data (Statistics Canada 2007).

As seasonal weather is an important contributing factor influencing patient arrivals (Mackay and Mensah 2004), the arrival rate is adjusted seasonally in our simulation. The patient arrival rate is approximately 15 % lower in the warm season (from May to October in Ontario) than in the cold season (from January to April and from November to December in Ontario), according to the reported CCN data (Alter et al. 2006).

Near 20 % of patients consider wait times when they select a hospital (Cardiac Care Network of Ontario 2005). Therefore, we assume that the probability that a patient considers the factor of wait times when selecting a hospital is relatively small and we set the probability* P*_*r*_ = 0.2 in our simulations.

According to the practice, patients can be categorized into two types, i.e.,* K* = 2. One type of patients is urgent, and another is non-urgent. According to the data reported by Alter et al. (2006, p. 71), the arrival rate of urgent patients versus that of non-urgent patients is set to 0.23:0.77. Urgent patients have a higher priority in receiving cardiac surgery services than non-urgent patients.

**Fig. 7:**
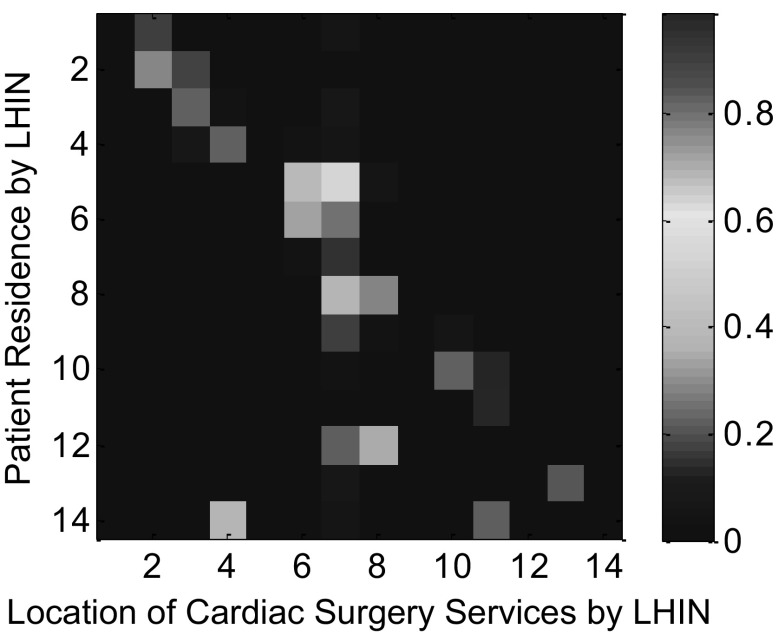
The distribution of operated cardiac surgery patients with respect to their residence by LHINs in the year of 2007–2008 in Ontario, Canada. This figure is adopted from the work of Tao and Liu (2013)

The values of exponential parameters (i.e.,* α*_*d*_, * α*_*r*_, and * α*_*w*_) are estimated by using the spatial pattern of real patient flows in 2007 (as shown in Fig. 7) (Cardiac Care Network of Ontario 2007). Based on our experiments, it has been found that when* α*_*d*_ = 4, * α*_*r*_ = 1, and * α*_*w*_ = 1, we can get relatively small values of mean and standard deviation of absolute errors. Here, the absolute error is defined as $|e_{ij}|=|\hat{a}_{ij}-\hat{a}^{\prime}_{ij}|$, where $e_{ij}$ is the error between the percentage of patients residing in LHIN* l*_*i*_ coming to hospitals in LHIN * l*_*j*_ in the year of 2007-2008 in Ontario, $\hat{a}_{ij}$, and the percentage of simulated patients that reside in LHIN * l*_*i*_ but visit LHIN * l*_*j*_ for services, $\hat{a}^{\prime}_{ij}$.

In accordance with the real-world monthly service utilization data from January 2005 to December 2006, we therefore set the total simulation time steps as 720 to represent the same period of time, i.e., 2 years. At each time step, the simulation repeats 1,000 times to get mean values of the number of generated patient entities and the number of served patients in a hospital. The key parameters as used in the simulation are summarized in Table 1.

**Table 1:** Key parameters as used in the simulation

Symbol	Meaning	Initialization value
*pop*_*i*_	The population size of a city/town	The population size for a specific city/town in 2006
*m*_*i*_	The patient-generation probability of a city/town in a cold season	The patient-generation probability for each city/town in the cold season of 2006 based on the work of Alter et al. (2006)
$m^{\prime}_i$	The patient-generation probability of a city/town in a warm season	0.85*m_*i*_
*d*_*ij*_	Distance from a city/town to a hospital	The average driving time calculated by Google Maps
*r*_*j*_	The number of physicians in a hospital	The number of physicians in a specific year (2005 or 2006) for a hospital
*w*_*j,r*_	The wait time information for a hospital at time round $\tau$	Average median wait times in the last quarter of 2004
*P*_*r*_	The probability of a patient considering the factor of wait times when selecting a hospital	0.2
*K*	The number of patient types	2 (i.e., urgent and non-urgent patients)
*μ*(*t*)	Average service rate of a hospital	The mean service rate in 2005 of a hospital
*queue*	The queue length of a hospital	The queue length at the end of the first quarter in 2005
*α*_*d*_	Sensitivity of a patient to the factor of distance	4
*α*_*r*_	Sensitivity of a patient to the factor of hospital resourcefulness	1
*α*_*w*_	Sensitivity of a patient to the factor of wait times	1
*b*_*j*_	The first service rate adjustment parameter for hospital *h*_*j*_	0.57
*g*_*j*_	The second service rate adjustment parameter for hospital *h*_*j*_	0.43
*t*	A unit of simulation time step	1 (day)
$\tau$	Time round, indicating the period of time to review the wait times in a hospital	1 (month)
$\hat{\tau }$	The number of time steps that are included in a time round $\tau$	30 time steps
$\tilde{\tau }$	The number of time steps that hospitals adjust the service rates* μ*(*t*)	1 week (i.e., five time steps)
*T*	The total simulation time steps	720 time steps

### Simulated patient arrivals and wait times

**Fig. 8:**
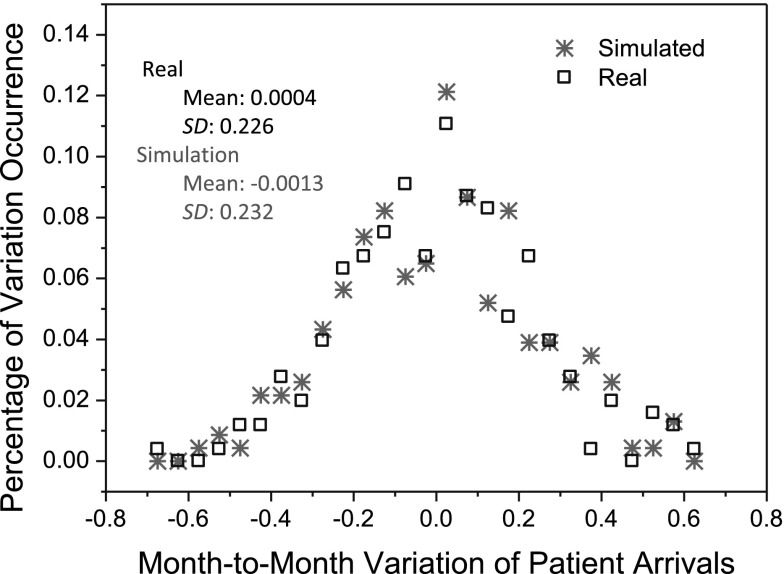
Distributions of variations in simulated and observed patient arrivals in cardiac surgery services.* SD* standard deviation

In this section, we examine the self-organized regularities in our synthetic cardiac surgery services. Figure 8 shows the comparison between the distribution of patient arrival variations in the real world (represented by black boxes in the figure) and that obtained from the simulation (represented by red stars in the figure). The simulation approximately reproduces the shape of the distribution of observed patient-arrival variations, shown in Fig. 8. The observed patient-arrival variations have a mean of 0.0004 and a standard deviation of 0.226, whereas the simulated patient-arrival variations have a mean of −0.0013 and a standard deviation of 0.232. The Kullback-Leibler (KL) divergence (Burnham and Anderson 2002), which measures the difference between the statistical distribution of simulated patient-arrival variations and that of real-world patient-arrival variations, is 0.14. The small value of KL divergence (0 means two distributions are identical while 1 means not (Burnham and Anderson 2002) implies that the distribution of patient-arrival variations as obtained from the simulation are close to that observed from the real world.

**Fig. 9:**
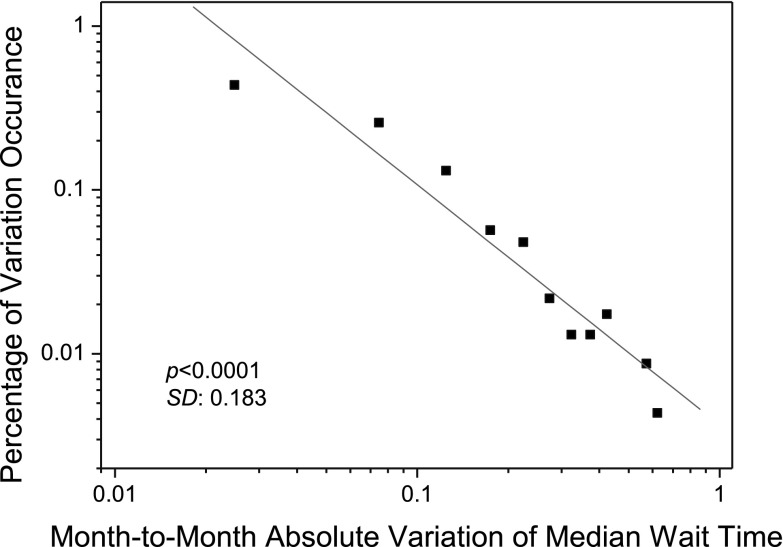
The distribution of simulated absolute wait time variations (by month) in cardiac surgery services. The distribution follows a power law with power of −1.47 (power-law test based on Clauset’s method (Clauset et al. 2009):* p* < 0.1; linear fitness (*red line*):* p* < 0.0001; standard deviation SD = 0.183). (Color figure online)

Figure 9 presents the statistical distribution of absolute variations of median wait time as obtained from our simulation. From Fig. 9, we can note that the absolute variations of median wait time in the simulation exhibit a power-law distribution with power of −1.47 (linear fitness: * p* < 0.0001; power-law test based on Clauset method (Clauset et al. 2009: * p* < 0.1)), while the power of the absolute variations of median wait time as observed in the actual practice is −1.36 (as illustrated in Fig. 3). This indicates that the synthetic cardiac surgery services are self-organizing in terms of wait times.

**Fig. 10:**
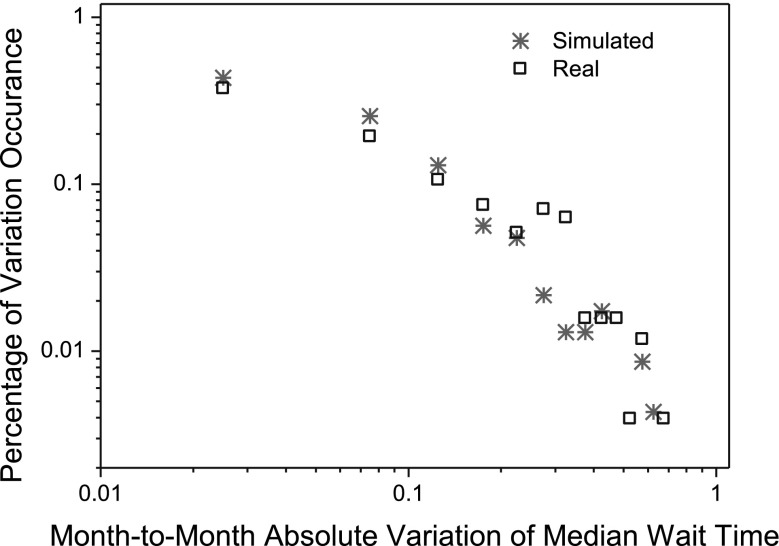
Distributions of simulated and real-world wait-time variations in cardiac surgery services

Figure 10 compares the statistical distribution of absolute variations in the median wait time obtained from our simulation to the distribution of the observed data. The KL divergence of the distribution of the simulated absolute wait-time variations (represented by red stars in the figure) from that of the observed absolute wait-time variations (represented by black boxes in the figure) is 0.1227. The small value of the KL divergence implies that the two distributions are similar.

## Discussion

### Causes of tempo-spatial patterns

Based on our AOC-CSS model and simulation-based experiments, we are able to characterize the self-organized regularities as observed in the real-world cardiac surgery services. This is partially due to the AW-loop as shown in Fig. 4.

**Fig. 11:**
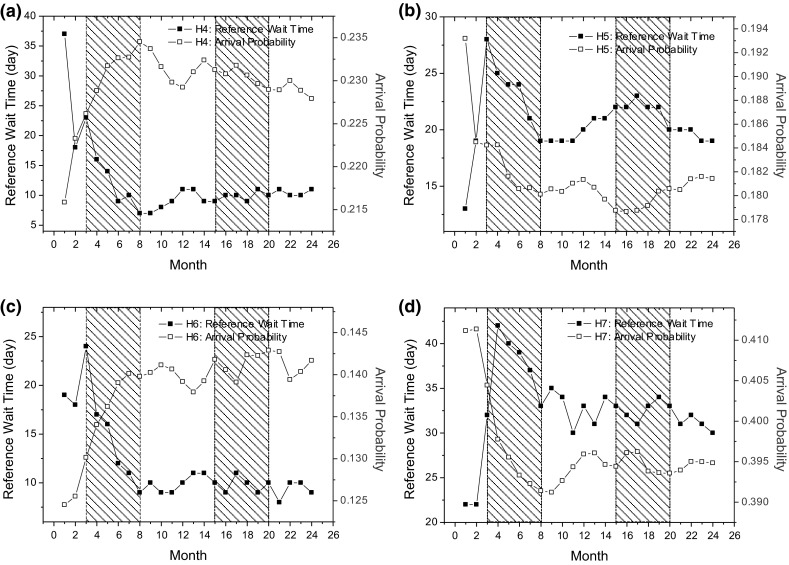
The dynamically-changing preferences of patients residing in the city of Brampton (in LHIN 5) to the four neighboring hospitals, i.e.,** a** H4, Trillium Health Centre.** b** H5, St. Michael’s Hospital.** c** H6, Sunnybrook Hospital.** d** H7, University Health Network. The* shaded areas* in this figure represent the warm seasons in Ontario, Canada

Let us take the city of Brampton, Ontario, as an example to illustrate the self-organizing process at an individual level. The four hospitals nearest to Brampton that offer cardiac surgery services are Trillium Health Centre (H4), St. Michael’s Hospital (H5), Sunnybrook Hospital (H6), and University Health Network (H7). The average driving times for patients living in Brampton to these hospitals are less than 0.7 h. Figure 11 presents the dynamically changing preferences of patients residing in Brampton to the four hospitals and shows that patients living in Brampton generally prefer H7, because the driving distances from Brampton to the four hospitals are almost the same, varying between 0.5 and 0.7 h, and H7 has more physicians than the other three hospitals. As the values for the factors of driving distance and hospital resourcefulness are not changed during the simulation, the changing wait times for the four hospital are the only cause of the dynamically changing arrival probabilities.

For instance, Fig. 11(d) shows that in the first two months, the arrival probabilities for patients living in Brampton to H7 are high, because the wait times in this hospital are short, at approximately 22 days. Due to the high arrival probabilities in the first two months, more patients will visit H7 than the other three hospitals, which will in turn result in longer wait times in H7. The wait time information for H7 is then released into the environment and is used by patients when they make hospital selection decisions in the third month. As a result, the arrival probability of patients living in Brampton to H7 in the third month will decrease. This self-regulating process is initiated by autonomous patient/GP entities according to their hospital selection behavioral rules and incorporates the AW-loop, potentially accounting for the observed self-organized regularities at a systems level.

Figure 11 also shows that the trends of the changes in arrival probabilities to the four hospitals are complementary. The increase in arrival probabilities to some of the hospitals in some months therefore accompanies the decrease in arrival probabilities to other hospitals. Due to the differences in the wait times in the four hospitals, a few patients may therefore transfer among the four hospitals to avoid a long wait. For instance, in the first warm season (from month 3 to month 8), the arrival probabilities to H4 and H6 increase because their reference wait times are less than 20 days, whereas the arrival probabilities to H5 and H7 decrease because their wait times are much longer than 20 days. It should be noted that although the arrival probabilities to H4 and H6 increase, the wait times in all four hospitals decrease in the first warm season. The number of patient arrivals in the warm season is smaller than in the cold season. As more patients may be willing to travel to H4 and H6 in the first warm season, the accumulated patient arrivals in the first warm season may result in the increase in wait times in the initial several months in the second cold season (from month 9 to month 12), which will in turn reduce the arrival probabilities for the two hospitals. With the same analysis process described above, we can explain the variations in the arrival probabilities and wait times for the four hospitals in the subsequent months.

### Wait time variations at different time scales

**Fig. 12:**
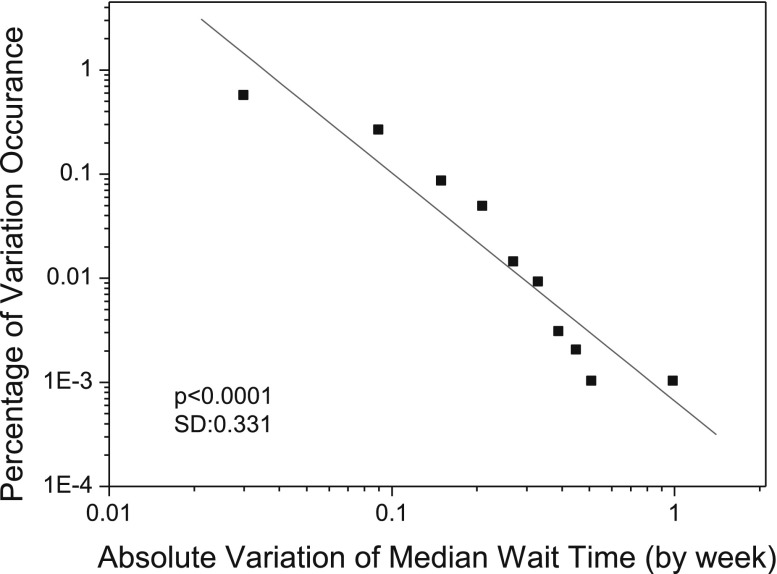
The distribution of simulated absolute wait time variations (calculated by week) in cardiac surgery services. The distribution follows a power law with power of −2.19 (power-law test based on Clauset’s method (Clauset et al. 2009):* p* < 0.1; linear fitness (*red line*):* p* < 0.0001; standard deviation SD = 0.331). (Color figure online)

**Fig. 13:**
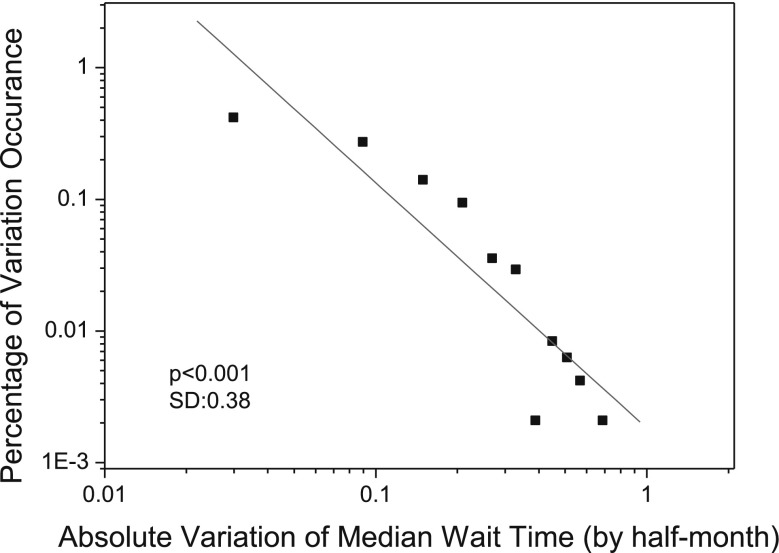
The distribution of simulated absolute wait time variations (calculated by half-month) in cardiac surgery services. The distribution follows a power law with power of −1.86 (power-law test based on Clauset’s method (Clauset et al. 2009): * p* < 0.1; linear fitness (*red line*): * p* < 0.001; standard deviation SD = 0.38). (Color figure online)

Figures 12 and 13 show the statistical distributions of absolute wait time variations that are calculated by week and by half-month, respectively. The power-law tests based on the Clauset’s method (Clauset et al. 2009) show that both of the absolute wait time variations as presented in the two figures fit power law distributions (power-law test:* p* < 0.1). The powers of the two statistical distributions are −2.19 and −1.86, respectively. This suggests that absolute wait time variations in different time scales are able to represent the self-organizing property of the cardiac care system in terms of wait times, such as by week, as shown in Fig. 12, by half-month, as shown in Fig. 13, and by month, as shown in Fig. 9.

### The probability for selecting DHW-rule, ${\mathbf {P}}_{{\mathbf {r}}}$

Table 2 presents the corresponding* p* values of power-law tests with respect to various* P*_*r*_ based on Clauset’s method (Clauset et al. 2009). According to Table 2, when there are few wait time-sensitive patients (e.g.,* P*_*r*_ = 0 or 0.1), the distribution of absolute wait time variations does not follow a power-law distribution, as the power-law test is not significant (* p* > 0.1). If most of the patients select hospitals without considering the wait time information, the AW-loop and the ASW-loop are absent. In other words, patient arrivals may not adapt to the dynamically changing wait times in hospitals.

**Table 2:** The* p* values of power-law tests for distributions of absolute wait time variations with respect to different* P*_*r*_

*P*_*i*_	1.0	0.9	0.8	0.7	0.6	0.5	0.4	0.3	0.2	0.1	0.0
>0.1	>0.1	>0.1	>0.1	>0.1	>0.1	>0.1	>0.1	<0.1	≤0.1	>0.1	>0.1

If $p\le 0.1$ as suggested by Clauset et al. (2009), the data for power-law fitness tests follows a power-law distribution.* P*_*r*_ is initialized to 0.2 in our simulations because near 20 % of surveyed patients in Ontario consider wait times when they select a hospital (Cardiac Care Network of Ontario 2005)

According to Table 2, when there is a relatively small probability that a patient considers wait times when choosing a hospital, e.g.,* P*_*r*_ = 0.2 or 0.3, the distribution of absolute variations in the median wait time follows a power-law distribution (*p* ≤ 0.1), suggesting that the system is self-regulating. This suggests that a small number of wait time-sensitive patients may result in the emergence of self-organized regularities.

However, when* P*_*r*_ becomes larger, for instance, * P*_*r*_ > 0.3, as shown in Table 2, the distributions of absolute wait time variations do not follow power-law distributions. The* p*-values of the power-law tests are all larger than 0.1. A large number of wait time-sensitive patients may therefore not result in a self-regulating healthcare service system, as the patient arrivals for each hospital may fluctuate highly if more patients are sensitive to the wait time information when they select hospitals.

### The number of time steps for releasing wait time information, ${\hat{\tau }}$

**Fig. 14:**
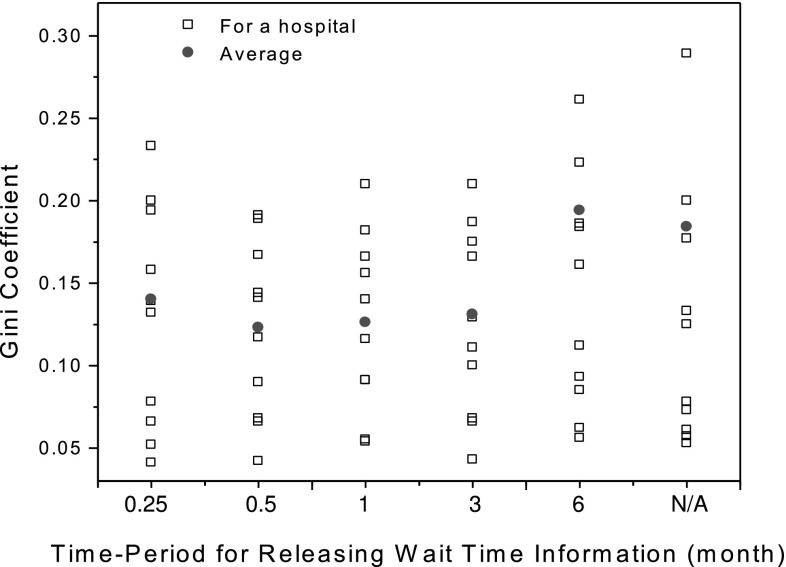
The Gini coefficients that measure the dispersion of wait times in a hospital with respect to different $\hat{\tau }$ for releasing wait time information.* Black box* a Gini coefficient of wait times for a hospital;* red dot* an average Gini coefficient of wait times for all hospitals. (Color figure online)

The parameter $\hat{\tau }$ is critical in that it determines the frequency for reviewing and releasing the wait time information to environment* E*. Figure 14 shows the Gini coefficients (Gakidou et al. 2000), which are utilized to measure the variations of wait times in a hospital, with respect to different $\hat{\tau }$. As denoted by the red dots in Fig. 14, reviewing the wait time information once every 0.5–3 months would reduce the Gini coefficient of wait times. This means that frequently updating the past wait time information may help regulate wait times in the healthcare service system. However, Fig. 14 also reveals that releasing the wait time information too frequently, e.g., once every week, may not decrease the extent of variations in wait times. This is potentially because the wait time information calculated within a small $\hat{\tau }$ may be biased, and thus is hard to regulate patient arrivals and wait times.

## Conclusion

In this paper, we have used an AOC-based modeling and simulation approach to characterizing self-organized regularities in cardiac surgery services. In particular, we have described three types of entities, i.e., patient, GP, and hospital, as well as the environment that they reside in and access information from. Based on the identified major impact factors of distance, hospital resourcefulness, wait times, as well as their interaction relationships and local feedback loops, we have derived three types of behavioral rules for patients to make mutual decisions with their GPs on hospital selection and hospitals to adaptively adjust their service rates.

Through simulation-based experiments, we have observed that the constructed AOC-CSS model produces a few regularities that are, more or less, similar to those found in the real-world cardiac surgery services. This indicates that the patient-GP mutual hospital selection behavior and its interrelationship with hospital wait times may account for the self-regulating service utilization. It also reveals that the AOC-based modeling approach provides a potentially effective means for explaining the self-organized regularities and investigating emergent phenomena in complex systems. In our future study, it would be promising to study the applications of the presented approach to other real-world complex healthcare services, so as to better understand how self-organized regularities at a systems level emerge from individuals’ collective behaviors and their closely coupled interactions.

## Footnotes

1
http://www.ccn.on.ca/ccn_public/FormsHome/HomePage.aspx

2
http://www.ccn.on.ca/ccn_public/uploadfiles/files/Patient%20Access%20Mgmnt%20diagram

3
https://maps.google.com/
